# Shedding Light on Inter-Individual Variability of Olfactory Circuits in *Drosophila*

**DOI:** 10.3389/fnbeh.2022.835680

**Published:** 2022-04-25

**Authors:** Karen Rihani, Silke Sachse

**Affiliations:** ^1^Research Group Olfactory Coding, Max Planck Institute for Chemical Ecology, Jena, Germany; ^2^Max Planck Center Next Generation Insect Chemical Ecology, Jena, Germany

**Keywords:** insect, antennal lobe, odor, sensory processing, olfactory behavior, neural circuits

## Abstract

Inter-individual differences in behavioral responses, anatomy or functional properties of neuronal populations of animals having the same genotype were for a long time disregarded. The majority of behavioral studies were conducted at a group level, and usually the mean behavior of all individuals was considered. Similarly, in neurophysiological studies, data were pooled and normalized from several individuals. This approach is mostly suited to map and characterize stereotyped neuronal properties between individuals, but lacks the ability to depict inter-individual variability regarding neuronal wiring or physiological characteristics. Recent studies have shown that behavioral biases and preferences to olfactory stimuli can vary significantly among individuals of the same genotype. The origin and the benefit of these diverse “personalities” is still unclear and needs to be further investigated. A perspective taken into account the inter-individual differences is needed to explore the cellular mechanisms underlying this phenomenon. This review focuses on olfaction in the vinegar fly *Drosophila melanogaster* and summarizes previous and recent studies on odor-guided behavior and the underlying olfactory circuits in the light of inter-individual variability. We address the morphological and physiological variabilities present at each layer of the olfactory circuitry and attempt to link them to individual olfactory behavior. Additionally, we discuss the factors that might influence individuality with regard to olfactory perception.

## Introduction

Researchers studying animal behavior are confronted with the diversity of behavioral outputs among individuals. Even individuals with nearly identical genotypes display different behavioral personalities. It is important to note that variability across individuals does not always reflect idiosyncratic behavior. A specific behavior is considered as a trait of individuality if it designates behavioral features that differ among conspecifics and persist over trials. This phenomenon has been described in humans (Johnson et al., 2009), rodents (Freund et al., 2013), fish (Vogt et al., 2008) and insects (Schuett et al., 2011) comprising various behaviors, such as startle, social, reproductive, locomotor, phototaxis, aggression as well as olfactory behaviors (Vogt et al., 2008; Schuett et al., 2011; Honegger and de Bivort, 2018). The vinegar fly *Drosophila melanogaster* represents a powerful genetic model organism to investigate variability among individuals. In fact, animals with the same genotype can be studied at a behavioral, physiological, anatomical and molecular level. Several studies that analyzed behavioral variability in *Drosophila* strongly contributed to our present knowledge regarding relevant brain regions and underlying genes that might be involved in idiosyncrasy (Kain et al., 2012; Ayroles et al., 2015; Buchanan et al., 2015; Honegger et al., 2020). Notably, the vinegar fly exhibits individual behaviors that persist over days in phototaxis (Kain et al., 2012), spontaneous locomotor biases (Buchanan et al., 2015), thermal preference (Kain et al., 2015), leg postural dynamics and locomotion (Todd et al., 2017), object-fixated locomotion (Linneweber et al., 2020), olfactory learning (Smith et al., 2021) and innate odor-guided behavior (Honegger et al., 2020). Even though individuality is present in every behavior and might shape the personalities of animals, it is rather disregarded and not taken into consideration in the final data presentation. Additionally, the link of individual differences between brain structures and physiology to the idiosyncratic behavior is still poorly understood. The comprehension of individual behavior and its relationship to brain structure and function will shed light on the strategies used by animals to differentiate themselves from others and allow them to adapt to environmental fluctuations. Individuality is a highly interesting phenomenon which gives important insight into how neural circuits develop and what internal as well as external factors are determining a behavioral output.

In this review we focus on the sense of smell of the vinegar fly, since this offers an ideal model system to study inter-individual variability. Over the last decades, numerous studies have identified the anatomical, molecular and genetic basis of the fly’s olfactory behavior (Harris, 1972; Venkatesh and Naresh Singh, 1984; Siddiqi, 1987; Stocker, 2001; Jones et al., 2007; Kwon et al., 2007; Vosshall and Stocker, 2007; Pask and Ray, 2016; Gomez-Diaz et al., 2018; Yan et al., 2020). Moreover, functional imaging as well as EM based connectomic studies have elucidated in great detail the associated brain circuits involved in the processing of olfactory information (Wang et al., 2003; Berck et al., 2016; Grabe et al., 2016, 2020; Horne et al., 2018; Zheng et al., 2018; Frechter et al., 2019; Bates et al., 2020; Marin et al., 2020). Such information will help us to highlight subjects to variability at the olfactory circuit level that will take this field a step further and decipher the observed differences in the behavioral output of different individuals.

**FIGURE 1:**
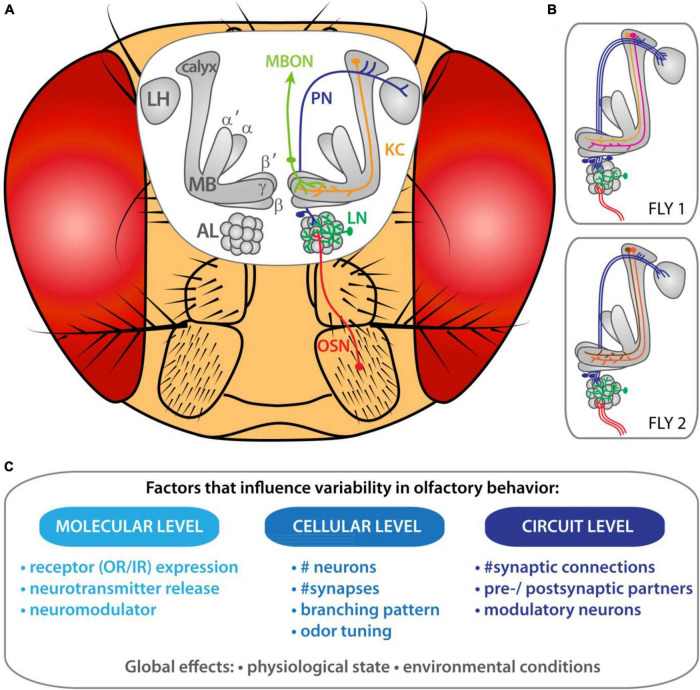
Inter-individual variability of olfactory circuits in *Drosophila melanogaster*. **(A)** Organization of the olfactory system in the vinegar fly. **(B)** Schematic representation of the morphological variabilities present at each layer of the olfactory circuit in two individual flies. **(C)** Factors that might impact olfactory personalities.

Flies detect odors with the help of olfactory sensory neurons (OSNs) present on the third antennal segment and the maxillary palps (Stocker, 1994; Figure 1A). These olfactory appendages are covered with sensilla and each sensillum houses between one to four OSNs (Venkatesh and Naresh Singh, 1984; De Bruyne et al., 2001). Each OSN expresses one specific chemosensory receptor from two gene families—odorant receptors (ORs) or ionotropic receptors (IRs)—in combination with not only one, but several co-receptors (i.e., Orco, Ir8a, Ir25a, and/or Ir76b) as recently shown (Task et al., 2020). All OSNs project their axons to the antennal lobe (AL) and converge upon one specific olfactory glomerulus (Clyne et al., 1999; Gao and Chess, 1999; Vosshall et al., 1999, 2000; Benton et al., 2009). A given odor activates different OSN classes and their respective glomeruli in a combinatorial manner (Grabe and Sachse, 2018). Interglomerular connections are provided by local interneurons (LNs) (Wilson and Laurent, 2005; Seki et al., 2010; Mohamed et al., 2019). Following pre-processing, the olfactory information is transferred to higher brain centers, such as the mushroom bodies (MB) and the lateral horn (LH), through olfactory projection neurons (PNs) (Jefferis et al., 2007; Lin et al., 2007; Fişek and Wilson, 2014). The LH is believed to primarily mediate innate behavior (e.g., De Belle and Heisenberg, 1994; Jefferis et al., 2007; Das Chakraborty and Sachse, 2021), while the MBs form olfactory associative memories (e.g., Heisenberg, 2003; Hige, 2018). The processed odors information is subsequently translated into a behavioral output.

In this review we would like to revisit the anatomical and functional features of the olfactory circuitry at different processing levels in *Drosophila* in the light of inter-individual variability and discuss what that might imply for individualized odor-guided behavior. A first step toward identifying the origins of inter-individual differences in odor-guided behavior in flies is to give an overview of the morphological and physiological variabilities present at each layer of the olfactory circuitry (Figure 1B). Furthermore, we will describe the factors that might support the emergence of olfactory personalities (Figure 1C). We also explore at what processing level connections and cellular properties become specific to each individual animal. Finally, the link between the differential connectivity in the olfactory circuit and odor-preference individualities is discussed.

## Factors That Influence Olfactory Individuality

Genetic and environmental traits together shape the individuality of animal behavior. Animals with similar genetic background adapt their gene expression to the available resources present in the environment (Honegger and de Bivort, 2018; Koyama et al., 2020). Even among individuals with the same genotype reared under the same environmental condition, differences in the phenotype were noted in genetic studies (Lin et al., 2016). Moreover, during the life course of flies, the expression of genes is plastic leading to changes in the individuality of an animal (Juneja et al., 2016; Lin et al., 2016).

Genes underlie transcriptional variation between individuals that influence different behavioral outputs (Jin et al., 2001). Studies showed variations in genes associated with olfactory perception in *Drosophila melanogaster*. Genetic variation in specific olfactory receptors or genes associated with neural development and the later processing in the central nervous system induces divergent odor guidance behavior among individuals of the same population (Richgels and Rollmann, 2012; Brown et al., 2013). This aspect is further discussed in the section “Variability at the level of olfactory sensory neurons.” The genotypic variation is also observed in other traits such as lifespan or morphological and anatomical structures (e.g., brain, wing, thorax, or eye size) (Carreira et al., 2016; Buchberger et al., 2021). Studies using the Drosophila Genetic Reference Panel (DGRP) have found the genetic origin involved in the variation between individuals expressing specific behaviors such as flight performance (Spierer et al., 2021), virgin egg retention (Akhund-Zade et al., 2017), aggressive behavior, immune response against pathogens (Guzman et al., 2021) as well as mating behavior (Gaertner et al., 2015).

In addition, researchers studied the implication of neuromodulators, such as serotonin or dopamine, with regard to inter-individual variability (Maloney, 2021). Interestingly, idiosyncrasy in olfactory behavior was reduced in a dose-dependent manner when the flies were fed on food containing the serotonin synthesis inhibitor alpha-methyltryptophan (Honegger et al., 2020). In contrast, activating the contralaterally projecting, serotonin-immunoreactive deutocerebral neurons (CSDn) had no effect on behavioral variability (Honegger et al., 2020). The former result is in line with a previous study showing that the neuromodulator serotonin affects the degree of idiosyncrasy in phototaxis behavior (Kain et al., 2012). The later result concerning the CSDn is consistent with the fact that synaptic connectivity of serotoninergic neurons is heterogeneous across glomeruli but stereotypic across individual flies (Coates et al., 2017). However, some degree of inter-individual variability has also been observed for the OSN-CSDn connectivity in a few glomeruli, as e.g., DA2 and VM2 (Coates et al., 2017, 2020). PNs and LNs express a diversity of serotonergic receptors that might be responsible for the effect of serotonin on the variability (Sizemore and Dacks, 2016). Additionally, also the neurotransmitter dopamine has been shown to have an impact on variability in odor-guided behavior. Dopaminergic neurons innervate the MB lobes in a compartmentalized manner and are crucial for associative learning (Aso et al., 2014). A mutation of the dopamine receptor gene (Dop1R1) induced lower variability in olfactory behavior than control flies, while a higher variability could be observed in flies that have been fed with the dopamine precursor L-DOPA (Honegger et al., 2020). In fact, Dop1R1 facilitates synaptic plasticity in the MBs (Kim et al., 2007; Qin et al., 2012). The effect of dopaminergic neurons in the MBs with regard to odor-tracking behavior was further investigated by Zolin et al. (2021). This study showed that dopamine can contribute to multiple forms of behavioral modulation by conveying motivational as well as instructive signals that shape current behavior and dictate future behavior through learning (Zolin et al., 2021). In general, neuromodulators seem to affect individuality at different levels: (i) variation in the amount of neuromodulation by differences in receptor expression, production of neuromodulators or activity in neuromodulatory neurons; (ii) alteration of circuit function to mask or accentuate circuit variability; (iii) facilitating plasticity of neural circuits. Hence, all these data suggest that serotonin and dopamine may control the degree of variability between individual flies (though not exclusively).

A recent study investigated biological mechanisms that affect variability/individuality with regard to locomotor behavior (de Bivort et al., 2021). A large data set of vinegar flies walking in Y-shaped mazes was evaluated by taking different biological mechanisms into consideration: the neuromodulator serotonin, white genotype, heterogametic sex and temperature. The results revealed that serotonin levels affected the variability of turn number, but had no strong effect that was consistent across behaviors. Notably, white genetic disruption correlated with small reductions in variability in turn bias and turn switchiness. Concerning the effect of sex on behavioral variability, male flies exhibited variability that was less in turn bias and greater in the number of turns as well as turn switchiness. On the other hand, high temperature significantly decreased the variability with regard to number of turns and turn switchiness but had no effect on turn bias variability. Overall, this study provided evidence that the effect on variability of the biological mechanism, as mentioned above, was behavior-dependent (de Bivort et al., 2021).

Developmental and growth conditions represent another important factor that has an influence on behavioral individuality. These variations are non-genetic and derive from stochastic microenvironment effects such as e.g., food sources. Interestingly, Honegger et al. (2020) demonstrated that an acute switch in the food diet from cornmeal/dextrose food to a commercial flake food led to an increase in variability of odor preference in flies. Environmental effects on behavioral variability were also investigated in other insects. For instance, the change of food quality does not impact the variability in risk-taking behavior of clonal pea aphids (Schuett et al., 2011), but influences the variability of risk-taking and activity in the beetle *Phaedon cochleariae* (Tremmel and Müller, 2013). Taking an example from the visual system of flies, Linneweber et al. (2020) showed that personalities in form of object orientation have a developmental origin. They demonstrated that stochastic variation of the axonal projections in the Dorsal Cluster Neurons within the medulla shapes the visual orientation of flies (Linneweber et al., 2020). In addition, the correlation between behavioral variability and the genetic diversity was investigated. Notably, the genetic background has no influence on the phenotypic variability with regard to visual orientation (Linneweber et al., 2020), while the degree of variability in locomotor handedness is itself genetically determined and thus heritable (Ayroles et al., 2015; Buchanan et al., 2015).

In the following section, we are discussing potential mechanisms underlying variability regarding olfactory behavior of flies. Further investigations are of course needed to verify whether these factors indeed influence the personalities observed. Exposure of flies to odors can influence the inter-individual variability in size and responsiveness of olfactory glomeruli in the AL. In fact, a 4 days exposure of flies to carbon dioxide or ethyl butyrate induced a significant increase in the volume of the responsive glomerulus (Sachse et al., 2007) in an odor-specific manner. In addition, the odor responses of second-order neurons (i.e., LNs and PNs) as well as the behavioral output were modulated by long-term odor exposure. Hence, the sensory environment can affect the morphology and physiology of the respective neurons in the first olfactory center, while it has been shown to shape the circuit organization in higher brain centers as well. For instance, the size, number and active zone density of microglomeruli (i.e., PN-KC synaptic contacts) in the MB calyx region is activity-dependent, since it is altered when the synaptic transmission is abolished in PNs (Kremer et al., 2010). These results were confirmed by another study showing that chronic deprivation of synaptic transmission of PNs reduced drastically the amplitudes of postsynaptic calcium transients of the AL as well as presynaptic calcium signaling in the MB calyx evoked by the odors methyl cyclohexanol, 3-octanol, apple and banana (Pech et al., 2015). Pech et al. (2015) also showed that prolonged exposure to apple reduces postsynaptic calcium signaling in the apple-responsive glomerulus DL5. Furthermore, the number of microglomeruli has been shown to be affected by associative olfactory learning (Baltruschat et al., 2021). These parameters (i.e., sensory deprivation, olfactory learning) should be tested in a paradigm that directly links the variabilities observed to olfactory personalities in individual flies.

Thus, epigenetic mechanisms, genetic variation, developmental growth and environmental conditions can shape the personality of an animal’s individuality (Figure 1C; Mollá-albaladejo and Sánchez-alcañiz, 2021). However, the variability of behavioral traits and their genetic and non-genetic origins need to be further studied to enlighten the evolution of personality traits.

## Variability at the Level of Olfactory Sensory Neurons

Flies rely on the detection of odor stimuli in the environment to find nutritive food, to avoid toxic compounds and to identify suitable ecological niches and mating partners. The first layer responsible of these tasks represents the OSNs expressing different types of receptors (i.e., ORs, IRs) as introduced above. It is conceivable that individuality in odor preferences derives from inter-individual differences at the peripheral olfactory organs, i.e., at the level of the first-order sensory neurons. Potentially, the expression levels as well as types of chemosensory receptors and/or neurotransmitter receptors might vary between individuals and impact the odor-evoked responses in OSNs (Figures 1B,C). For instance, odor-guided perceptions among *Drosophila* individuals of the same population was linked to genetic variation in ORs (Or22a/Or22b, Or35a, and Or47a) (Richgels and Rollmann, 2012). Another study revealed the effect of genes associated with neural development and the later processing in the central nervous system on variation in odor preference to 2,3-butanedione (Brown et al., 2013). However, the idea that variations in the expression levels of olfactory receptors and neural developmental genes enhance variability should be further investigated to find evidence for morphological or physiological changes in olfactory responses of afferent neurons.

The axons of OSNs converge onto a discrete glomerulus within the AL in the brain (Figure 1A). Notably, the number of OSNs innervating a given glomerulus varies across flies (Grabe et al., 2016), and structural variations in synaptic connections between OSNs and PNs have been identified (Tobin et al., 2017). A recent study tackled the dynamic of the cellular processes by which OSNs target axons precisely to a specific glomerulus in the ipsi- and contralateral AL (Li et al., 2021). During that process, OSN axons first form multiple ipsilateral branches, while only those branches that are close to their eventual glomerular target will be stabilized later on. The precise dynamic state of the branches (extending, retracting and stationary) varies between individuals (Li et al., 2021). One possibility is that the number of branches and therefore the strength of the diverse synaptic connections varies between individuals, which would represent an additional factor to facilitate individualization. However, the influence of these developmental differences on the variability of olfactory responses is unknown and needs to be further explored.

A recent study showed that certain neuronal populations of the olfactory circuit are predictive for individual behavioral responses (Churgin et al., 2021). Based on two-photon imaging measurements paired with behavioral assays, Churgin et al. (2021) built a model and found that idiosyncratic calcium dynamics as well as presynaptic densities of OSNs could predict the odor preference of flies. Furthermore, Churgin et al. (2021) investigated the capacity of predicting individual behavioral responses from the calcium dynamics in PNs which will be discussed in the following section.

Second-, third- and higher-order neurons are shown to exhibit morphological variations between individuals with regard to wiring and synaptic connectivity and could therefore provide the neural substrate in the brain to support individualities in odor-guided behavior. We will summarize in the following section the so far described inter-individual variabilities at the different olfactory processing levels.

## Variability at the Level of the Antennal Lobe

The *Drosophila* AL possesses 58 identifiable glomeruli (Grabe et al., 2015). The glomeruli are organized in a consistent spatial pattern and have genetically determined shapes, positions and sizes across individuals as well as stereotyped OSN inputs and PN outputs (Laissue et al., 1999; Couto et al., 2005; Fishilevich and Vosshall, 2005; Jefferis et al., 2007; Lin et al., 2007; Grabe et al., 2015). LNs innervate the AL and provide intra- and inter-glomerular inhibition (i.e., presynaptic inhibition of OSNs, feedforward inhibition onto PNs) (Wilson and Mainen, 2006; Olsen and Wilson, 2008; Root et al., 2008; Mohamed et al., 2019). Highly comprehensive characterization of LNs in the *Drosophila* AL was established previously and has led to the categorization of LNs based on neurotransmitter profiles, connectivity, as well as morphological and physiological properties (Chou et al., 2010; Seki et al., 2010). Hence, different classes of LNs exhibit morphological and physiological differences. Moreover, a considerable variability in the density of arborizations and thicknesses of their processes is present within each category. This finding raises the following question: Does the variability of the same LNs across different individual flies represent the origin of the LN’s morphological and physiological diversity? Indeed, the number of distinct innervation patterns in ipsilaterally projecting LNs exceeds the estimated total number of ipsilaterally projecting LNs within an individual AL. In other words, there are far more anatomical classes of LNs across individuals than there are LNs in an individual fly brain (Chou et al., 2010). This finding indicates that LN arborization patterns are not completely stereotyped across flies and seem to be rather unique in each fly (Figure 1B). Furthermore, physiological studies on specific GAL4 lines that label a small population of LNs identified diverse functional properties. Nevertheless, the properties of these LNs are not drawn randomly from the entire distribution of LN properties. In fact, odor response properties, i.e., mean, maximum odor-evoked and spontaneous firing rates were less variable across small populations of LNs than across all LNs. All these data indicate that the coarse properties of these neurons might be genetically pre-programmed, but do also reflect developmental plasticity and sensory experience (Chou et al., 2010). Along that line, a recent study demonstrated that activating or inhibiting different populations of LNs reduced variability in olfactory behavior (Honegger et al., 2020).

The olfactory information formed at the level of the AL is sent to higher brain centers *via* PNs. PNs extend their dendrites into a single glomerulus and project their axons to innervate the LH and MB. The olfactory system of the fly possesses two types of PNs: uniglomerular PNs (uPNs) that innervate a single glomerulus, and multiglomerular PNs (mPNs) that branch within subsets of glomeruli. uPNs have been intensively studied and could be classified due to their specific odor response profiles as well as their steretyped branching patterns in the AL and LH (Marin et al., 2002; Wong et al., 2002; Wang et al., 2003; Wilson et al., 2004; Bates et al., 2020), while mPNs could only be classified into two broad categories based on their innervated glomeruli in the AL (Strutz et al., 2014). However, the number of uPNs innervating a given glomerulus is not stereotypic and varies across animals (Grabe et al., 2016), while we do not have this information about mPNs. Moreover, recordings of odor responses of uPNs innervating specific glomeruli reveal some degree of inter-individual variability (Honegger et al., 2020). However, functional and anatomical clustering among the uPN population is still possible despite their inter-individual differences, since the targeted glomerulus is strictly conserved among individuals (Jefferis et al., 2007; Murthy et al., 2008). Nevertheless, the differences across odor-evoked PN responses might still reflect and explain the individuality observed in odor preferences: Honegger et al. (2020) characterized odor responses of dozens of animals to a dozen odors, in PNs of the AL. They observed that responses of some glomeruli were very different across individuals, but consistent across multiple presentations of the same odor within an individual. Moreover, this study revealed that the within-fly responses were closer correlated than between-fly responses. All these data demonstrate that PN responses to odors differ significantly across individuals and are idiosyncratic (Honegger et al., 2020). A recent study further investigated the link between neuronal responses and individual odor preferences (Churgin et al., 2021). Churgin et al. (2021) could predict the idiosyncratic odor preference of flies using the calcium imaging responses of PNs. Overall, the results of this study suggest that physiological variations in PN responses might be driven by the developmental variability of OSN populations leading to individuality in odor preference behavior.

Similar to *Drosophila*, also other insects exhibit inter-individual variabilities in their olfactory circuits with regard to neuronal wiring, synaptic connectivity as well as anatomical features. The olfactory glomeruli of the noctuid moth *Spodoptera littoralis* can be clearly identified in different ALs of different individuals. However, several types of biological variability were observed here as well: For instance, some glomeruli were lacking in some individuals which indicates either the absence of the corresponding OR/IR/OSN type or a mistargeting to another glomerulus during development. Contrary to *Drosophila*, the AL of *Spodoptera littoralis* varies in its global shape which leads to changes in the spatial location of the glomeruli. Interestingly, several other moth species also exhibit variations in the number and size of their glomeruli in the AL, such as *Mamestra brassicae* (Rospars, 1983), *Manduca sexta* (Rospars and Hildebrand, 1992, 2000), and *Bombyx mori* (Rospars and Chambille, 1981; Rospars, 1983; Kazawa et al., 2009).

## Variability at Higher Brain Centers – the Mushroom Body Level

The MBs are composed of approximately 2,500 intrinsic neurons known as Kenyon cells (KCs). The KC’s dendrites form the MB calyx while their axonal fibers form the output lobes of the MB (γ, α′/β′, α/β lobes). The main olfactory inputs received by the MB calyx are provided by PNs from the AL. Anatomical and physiological studies showed that on average 6–8 PNs innervate each KC (Caron et al., 2013; Gruntman and Turner, 2013; Bates et al., 2020). Caron et al. (2013) characterized the glomerular origin of those PNs that converge onto one KC by photolabeling individual KCs. Their study showed that the majority of individual KCs integrates random and not stereotyped combinations of glomerular inputs (Figure 1B). Notably, neither the odor tuning nor anatomical features or developmental origins dictate a specific organization of the glomerular inputs to an individual KC. Moreover, electrophysiological responses of KCs to different odors in a fly line labeling 23 α/β neurons revealed distinct odor response profiles of KCs among individuals (Murthy et al., 2008). It is well established that learning and experience-dependent behavior rely on the plasticity and the described random organization of the MBs (Bilz et al., 2020). The inter-individual variability of KC responses and the random PN-KC connectivity facilitates flexibility of the olfactory system to adjust to environmental changes, previous experience and internal state. However, these data raise the question whether the random organization of glomerular inputs to the MBs could also account for the observed individuality in odor-driven behavior. Indeed, a given odor will activate different sets of KCs in different flies and trigger behavioral outputs that are likely to vary across individuals (Figure 1B).

One specific feature of the MB circuit is that the output to further brain areas is conveyed by solely 34 MB output neurons (MBONs) that can be categorized into 21 cell types. Dendrites of each MBON type innervate distinct subregions of the MB lobes. These numbers reflect the heavy convergence from the KCs onto MBONs (Tanaka et al., 2008; Mao and Davis, 2009; Aso et al., 2014). Many studies have characterized odor-evoked responses of MBONs, which usually normalize and average the measured odor responses between flies, leading to the loss of information concerning inter-individual variability as mentioned above. In contrast, the study by Hige et al. (2015) clearly emphasizes variability of odor responses of MBONs across flies by demonstrating that some MBONs with uniquely identifiable anatomy have diverse tuning properties in different animals. Interestingly, across all MBONs, the α2sc neurons exhibit the greatest amount of variability (Hige et al., 2015), a MBON type that is required for the retrieval of aversive olfactory memories (Séjourné et al., 2011). However, the odor tuning patterns of MBON-α2sc from the two brain hemispheres of the same animal are strikingly similar indicating that processes coordinated across both hemispheres must dictate the tuning patterns of this MBON type. To assess whether the variable tuning properties derive from fluctuating levels of population activity in KCs or by the functional connectivity between KCs and MBONs, Hige et al. (2015) demonstrated that the KC-MBON-α2sc connection differs among individuals, while the calcium responses in the KC axon bundle were similar from fly to fly. Hence, the individual-specific connectivity of MBON-α2sc enables the neurons to extract different information among individuals, even from presynaptic KCs that exhibit a similar overall population tuning. Moreover, the diversity in wiring across flies might be caused by synaptic plasticity, since mutants of the rutabaga gene encoding a calcium-dependent adenylyl cyclase required for learning, reduced (but did not abolish) the tuning variability of MBON-α2sc across flies (Hige et al., 2015). Adaptive plasticity of calcium activity of MBONs was also reported in a recent study (Hancock et al., 2022). These findings suggest that elements implicated in learning processes and plasticity also influence the variability across flies. Hence, individualized coordination of tuning observed at the KC-MBON level might represent one of the origins of individuality in olfactory responses. However, additional behavioral experiments are necessary to provide the link between plasticity and individuality.

## Variability at Higher Brain Centers – the Lateral Horn Level

The LH represents a higher-order brain center that processes different sensory modalities including olfactory information (Frechter et al., 2019; Das Chakraborty and Sachse, 2021). Several studies have documented the role of the LH with regard to innate behavioral responses by encoding hedonic valence to odor cues, while the LH is also processing learned responses to previously encountered odors (Strutz et al., 2014; Dolan et al., 2019). The spatial organization of the LH is determined by the position of the PN axonal terminals that either directly project from the AL (most of mPNs) or that relay the olfactory information from the AL *via* the MBs (all uPNs and some mPNs) (Li et al., 2021). Comprehensive maps of higher olfactory centers of *Drosophila* reported in previous studies revealed a clear stereotypy of the branching patterns of PN axons in the LH (Marin et al., 2002; Wong et al., 2002; Jefferis et al., 2007). So far, the LH connectivity is less well understood than the MB circuitry (Das Chakraborty and Sachse, 2021). LH neurons (LHN) could be classified based on morphological, neurotransmitter and polarity information using the EM connectomic dataset as well as their odor response properties (Jeanne et al., 2018; Dolan et al., 2019; Frechter et al., 2019). However, some functional cell types exhibited a high degree of variability in their odor responses and were difficult to classify (Frechter et al., 2019). Moreover, recent analyses of the EM-based connectomics data showed that the PN input to LHNs of the same cell type can vary (Dolan et al., 2018; Jeanne et al., 2018). The origin of this response variability could either result from differences in the number or strength of inputs to that cell type across animals or just experimental factors. The latter suggestion was excluded by the study of Frechter et al. (2019) by providing evidence that no apparent relationship between cell-recording parameters (i.e., cell capacitance, membrane/pipette resistance) and the strength of the response could be found. Additionally, taking together all the recent advances in characterizing the cellular composition of the LH and analyzing the connectivity to PNs of the AL, it is very likely that synaptic partners are variable among individuals. Indeed, some LHNs receive synaptic inputs from glomeruli that differ between flies and even between both brain hemispheres (Cachero et al., 2020). These findings could either result from technical issues or reflect biological variability at the level of the PN-LHN connections (Cachero et al., 2020). However, the impact of these variable connections on inter-individual differences in odor-guided behavior in flies is so far unknown and requires the analysis of circuit elements in large numbers of individuals.

Overall, the morphological and physiological differences at each level of the olfactory circuitry probably contribute to the individuality seen in olfactory behavior. It is most likely the combination of all these differences between individuals that shapes a specific olfactory behavioral output. Hence, the diverse connectivity of the olfactory circuit optimizes its ability to respond appropriately to a rich array of olfactory experiences and a changing environment.

## Variabilities Between Brain Hemispheres

The majority of OSNs in *Drosophila* projects from the antennae bilaterally to both brain hemispheres by collaterals passing *via* the antennal commissure (Stocker et al., 1990; Couto et al., 2005). Nevertheless, the connectivity of OSNs between the brain hemispheres are diverse (Tobin et al., 2017). Neuronal tracing from serial EM sections showed that the number of PNs in glomerulus DM6 varies between two and four, and PN counts are often different between the right and left side. In fact, the right brain hemisphere possesses larger dendritic path length and a higher number of OSN synapses (Tobin et al., 2017). Multiglomerular neuron synapses of LNs and mPNs and presynaptic contacts of uPNs were also in greater numbers on the right than on the left side (Tobin et al., 2017). Moreover, Bates et al. (2020) explored the numerical stereotypy of 58 uniglomerular PN (uPNs) types across both hemispheres and revealed that the uPN number is twice as numerous on the left side in glomerulus VA1d.

In addition, asymmetric odor stimulation has been shown to evoke distinct activation in the left and right brain hemisphere as a result of contralateral inhibition (Mohamed et al., 2019). It could be shown that odor responses in a specific cluster of third-order LHNs, so-called ventrolateral protocerebrum neurons (VLPn) were suppressed by presynaptic LHNs when an odor was presented to the contralateral side. Thus, a lateralized odor stimulus is distinctively detected by higher-order neurons through contralateral inhibition leading to an enhanced perception of odor concentration gradients between both brain hemispheres (Mohamed et al., 2019). Hence, also variability with regard to odor lateralization between flies should be taken into consideration and quantified, since it is conceivable that this could differ between animals and might be another factor for individuality of odor preference behavior.

## Concluding Remarks

For long, researchers believed that odor responses are highly stereotypic across different individuals of the same species and that the variability in animal behavior is just due to limitations in methodological approaches. Researchers also considered that the majority of the quantitative differences might be the product of noisy developmental processes and thus not relevant. However, this idea should be re-evaluated since various recent studies have shown that flies, similar to other animals, exhibit an individualized perception of odors (Thomas-Danguin et al., 2014; Trimmer et al., 2019; Honegger et al., 2020; Kermen et al., 2020; Ruser et al., 2021). These findings reveal the genetic sources of variations and should change our concept about the insect brain and its reproducibility of putative “hard-wired” properties. Moreover, studies on inter-individual differences in the neuronal wiring of other modalities (vision, locomotion, etc.) (Vogt et al., 2008; Schuett et al., 2011; Honegger and de Bivort, 2018), enlighten us on the presence of non-genetic variability that has an effect on the individuality in animal behavior. In this review, we describe morphological and physiological variabilities that occur in the olfactory circuit between individual flies. The possible link between genetic or environmental factors and odor-preference individualities is also discussed, but still needs to be proven. Potential factors that would cause variable behavioral responses and support “olfactory personalities” are mentioned and discussed as well. This review raises two questions: First, are the occurring variations at the molecular, cellular and circuit level arbitrary or do they facilitate potential adaptations of the brain to environmental fluctuations? Second, what might be the benefit for the animal’s fitness and survival? One could argue that it is more costly for animals to preserve structure and function of their neuronal circuits across individuals, since the biophysics and development processes need to be constrained that build and maintain biological systems. In addition, during learning, brain centers responsible for assigning context- specific values take advantage of random and individualistic connectivity patterns as shown for the MBs (Caron et al., 2013; Hiesinger and Hassan, 2018; Zheng et al., 2018). This variation is also beneficial for innate behavior, as it allows an animal to adapt to unpredictable environmental conditions and fluctuations (Honegger and de Bivort, 2018).

Addressing the origin and significance of variable connectivity throughout the nervous system will increase our understanding of personality variations. This aspect will require analysis of circuit elements in a large number of individuals of a given species. To conclude, it can be stated that the variability throughout the olfactory system supports odor-preference individualities.

## Author Contributions

KR and SS conceived and wrote the review. Both authors contributed to the article and approved the submitted version.

## Conflict of Interest

The authors declare that the research was conducted in the absence of any commercial or financial relationships that could be construed as a potential conflict of interest.

## Publisher’s Note

All claims expressed in this article are solely those of the authors and do not necessarily represent those of their affiliated organizations, or those of the publisher, the editors and the reviewers. Any product that may be evaluated in this article, or claim that may be made by its manufacturer, is not guaranteed or endorsed by the publisher.
